# Supplementary figures and images for: Functional annotation of the animal genomes: An integrated annotation resource for the horse
Source: PLoS Genet. 2023 Mar 2;19(3):e1010468. doi: 10.1371/journal.pgen.1010468 (PMC10013926; doi:10.1371/journal.pgen.1010468)

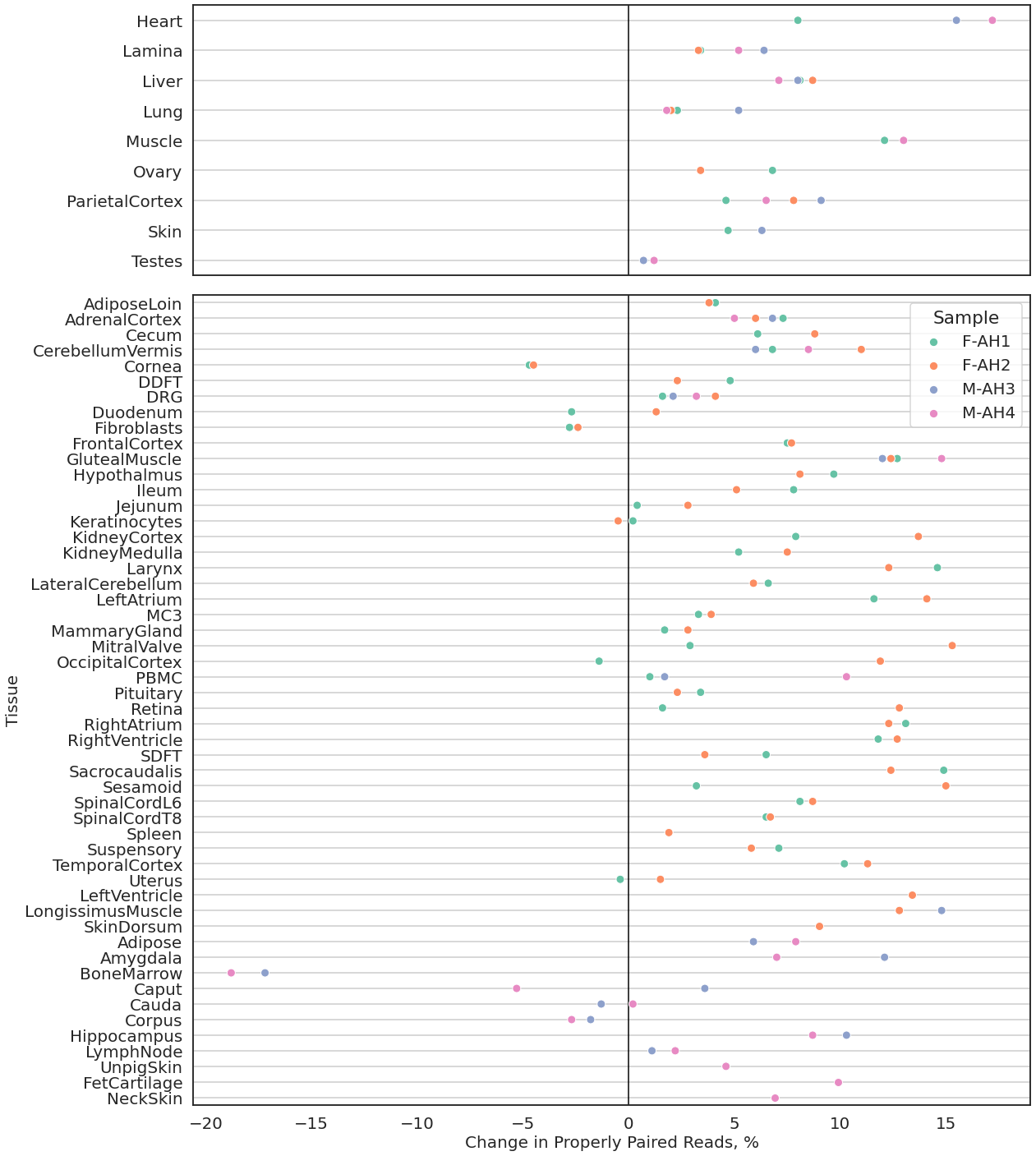

Supplement: S1 Fig — Changes in percentages of properly paired reads aligned to combined Iso-seq transcriptome when compared to Ensembl or RefSeq transcriptomes, whichever has higher percentage. (TIF) [file pgen.1010468.s001.tif]

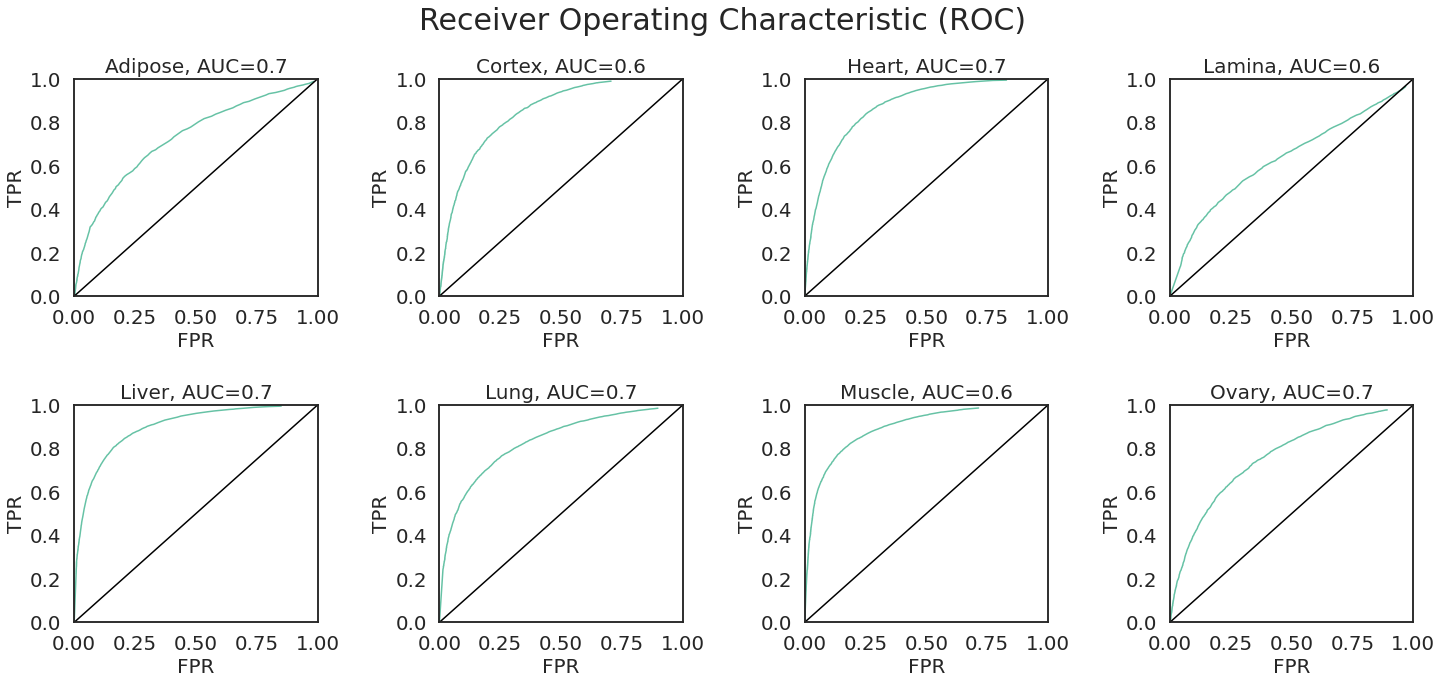

Supplement: S2 Fig — Receiver Operating Characteristics (ROC) of eight tissues whose ATAC-seq peaks were validated by Histone ChIP-seq data (TIF) [file pgen.1010468.s002.tif]

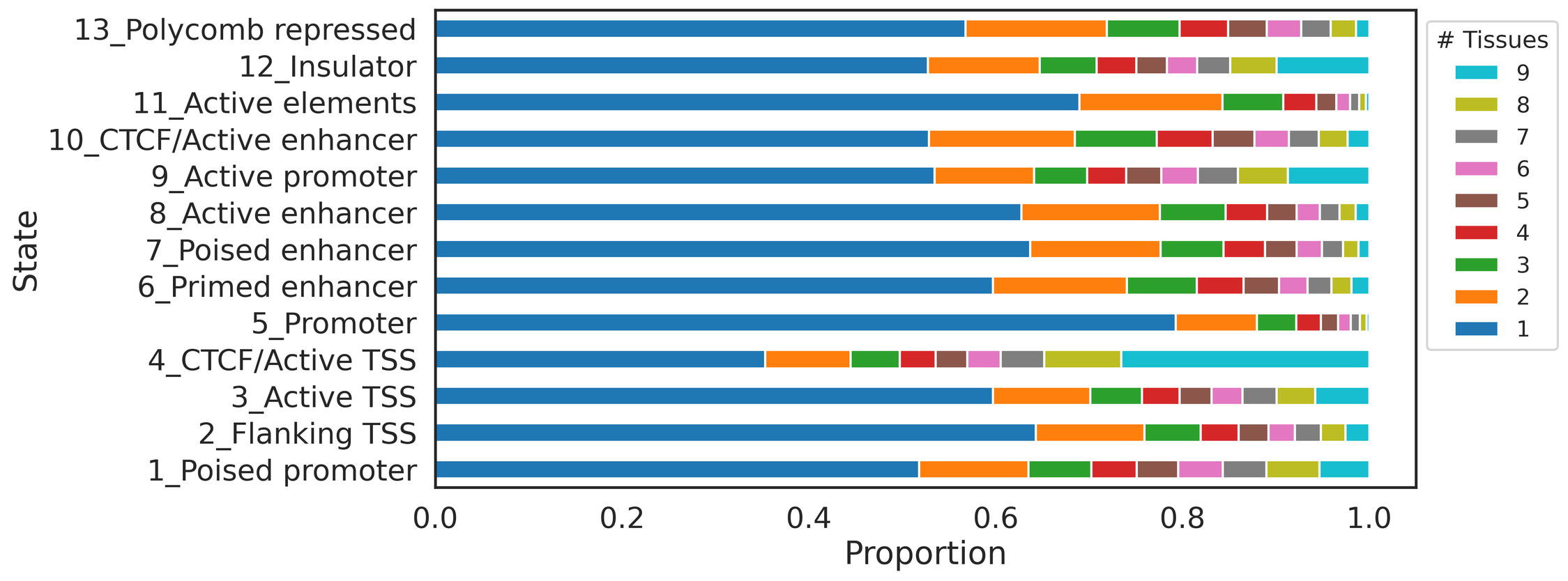

Supplement: S3 Fig — The proportion of segments from each state that were identified in different numbers of tissues. (TIF) [file pgen.1010468.s003.tif]

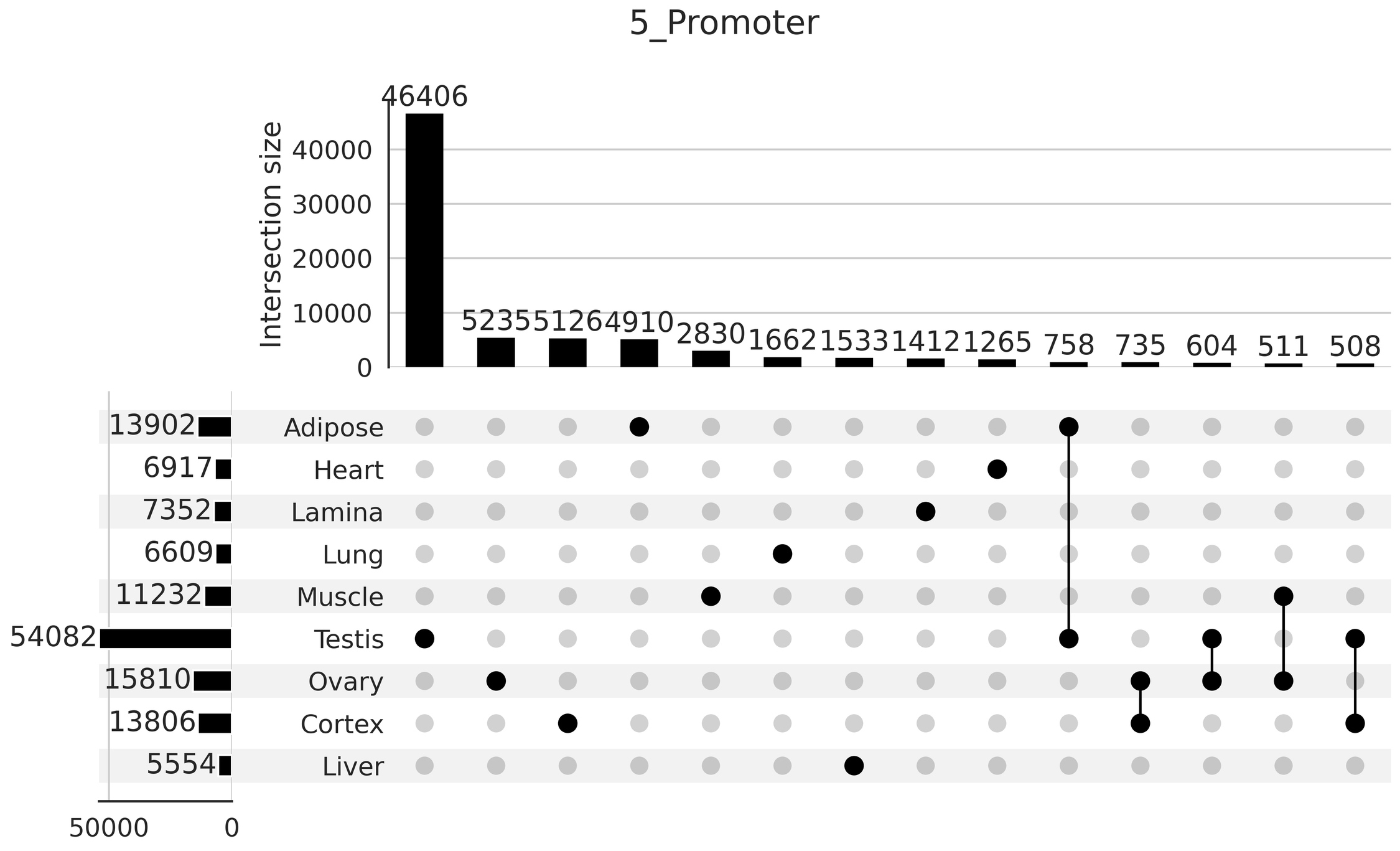

Supplement: S4 Fig — Intersection plot showing number of segments annotated as promoter state (state 5) unique to each tissue and shared across tissues. Top: bar plot indicates sizes of each intersection; Bottom right: each column denotes a unique set of peaks where filled dots indicat that peaks in this set were found in the corresponding tissue; Bottom left: bar plot indicates number of segments annotated as promoter state (state 5) in each tissue. (TIF) [file pgen.1010468.s004.tif]

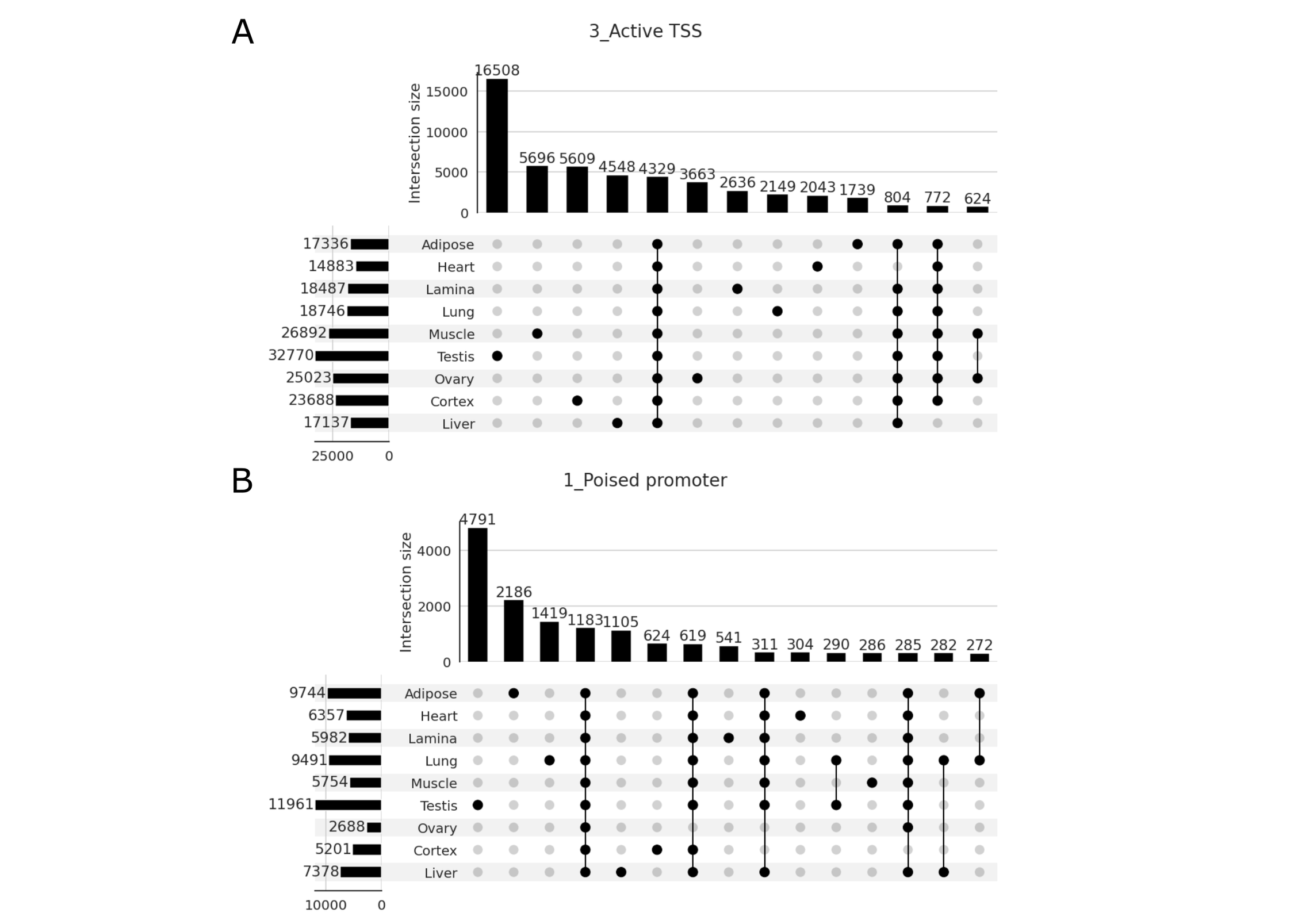

Supplement: S5 Fig — Intersection plots showing number of segments annotated as (A) CTCF-less active TSS state (state 3) and (B) poised promoter state (state 1) unique to each tissue and shared across tissues. Top: bar plot indicates sizes of each intersection; Bottom right: each column denotes a unique set of peaks where filled dots indicate that peaks in this intersection were found in the corresponding tissue; Bottom left: bar plot indicates number of segments annotated as (A) CTCF-less active TSS state (state 3) or (B) poised promoter state (state 1) in each tissue. (TIF) [file pgen.1010468.s005.tif]

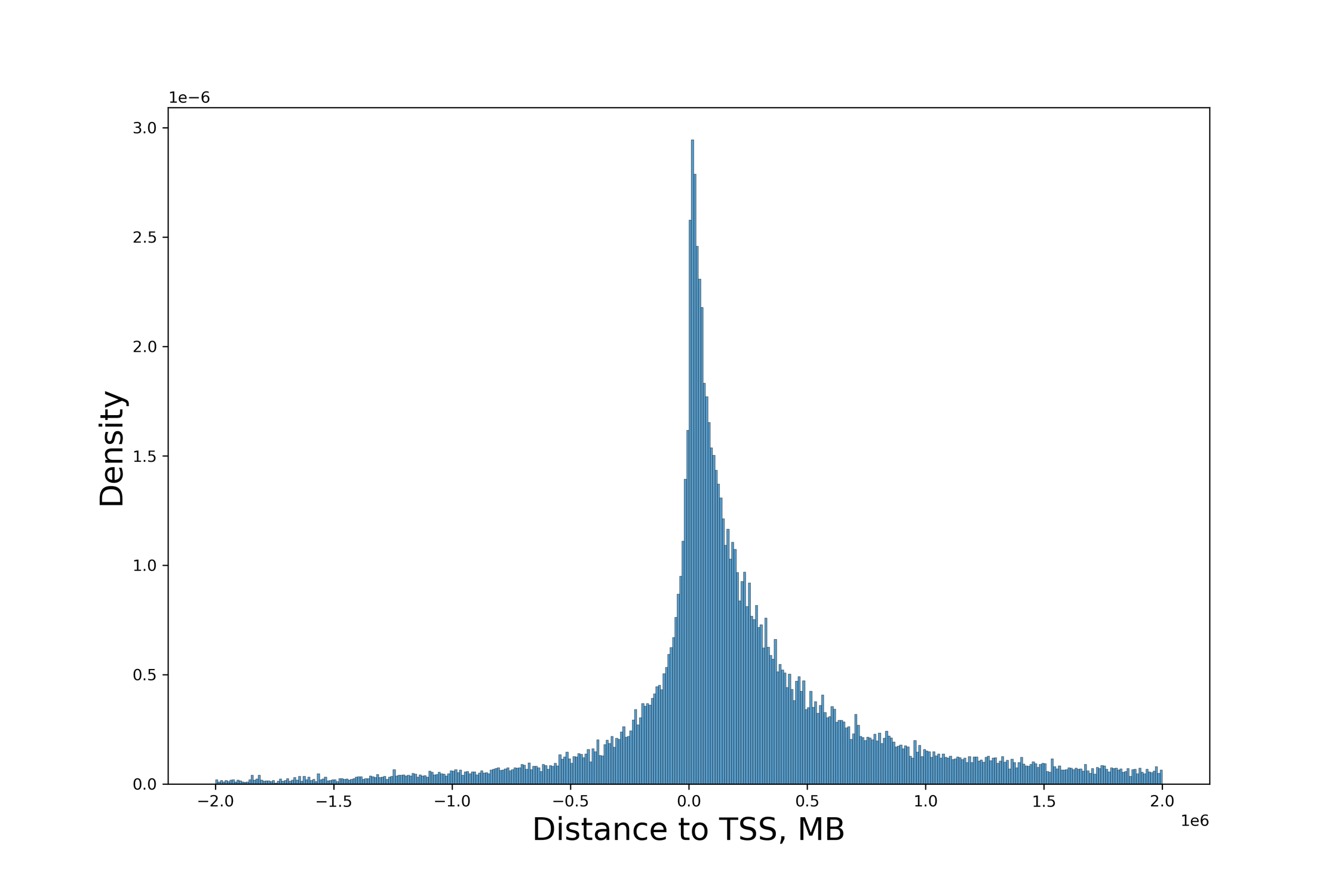

Supplement: S6 Fig — Density plot of distances from intergenic REs to their target genes’ TSS. Negative distance denotes RE being upstream of target TSS. Median absolute distance: 200 Kb. (TIF) [file pgen.1010468.s006.tif]
