# Supplementary material for: Functional annotation of the animal genomes: An integrated annotation resource for the horse
Source: PLoS Genet. 2023 Mar 2;19(3):e1010468. doi: 10.1371/journal.pgen.1010468 (PMC10013926; doi:10.1371/journal.pgen.1010468)
Supplement: Attachment — Submitted filename: Reviews.docx [file pgen.1010468.s015.docx]

Reviewer's Responses to Questions

**Comments to the Authors:
Please note here if the review is uploaded as an attachment.**

Reviewer #1: The manuscript presents the first comprehensive Iso-seq transcriptome of the horse. The authors centered around high-quality full-length reads from RNA Iso-seq and used abundant short-read RNA-seq data to refine the annotation of splice junctions and transcription start and termination sites. As a result, the equine transcriptome was improved by adding 39,625 novel transcripts, identifying 84,613 candidate cis regulatory element-gene pairs, and determining gene-isoform ratio of 4.2. As such, the manuscript delivers an awaited and integral resource for the studies of various aspects of equine biology and health by many research groups worldwide.

Comments:
The experimental data presented is of high-quality, the manuscript is well-written, concise and effectively integrates an extensive amount of functional annotation data across the horse genome. I do not have any critical comments and I am looking forward to see this manuscript published. There are just a few minor technical issues to mention:

Thank you for your comments. Please find our specific responses below, with line numbers used in the “simple markup” Word document format.

• Page 14, line 247: remove ‘and’ after (state 4)

Thank you for pointing out this typo. We have revised accordingly:

Line 258-259: “CTCF bound active TSS state (state 4) and showed a 59.4-fold enrichment around TSS of highly expressed (TPM ≥ 1) genes”

• Figure 1: for the ease of understanding, please explain in the legends the acronyms FSM and ISM (even though they are explained in the text)

We have revised the figure legend for clarity:

Line 657-660: “(D) Distribution of mapping rates against Iso-seq transcriptome vs. Ensembl or RefSeq transcriptome across 57 RNA-seq samples. FSM: full-splice match; ISM: incomplete-splice match; TTS: transcription start site; TSS: transcription termination site”

• Figure 4 right: the figure has ‘uniquitous’, while the legend says ‘ubiquitous’. Please correct.

Thank you for pointing out this typo. We have revised accordingly. See Figure 4

Reviewer #2: This paper presents a nice contribution to the FAANG international action regarding the annotation of the horse genome. RNA-seq and ATAC-seq experiments were performed on samples from 9 target tissues to identify transcriptomes and chromatin accessibility regions. Previously generated ChIP-seq data was integrated to complement the analysis. The resulting annotation includes gene models, open chromatin regions, Cis Regulatory Elements and epigenetic states of the chromatin. Results are provided as a custom track in the UCSC genome browser for visualization and download.

The article is correctly written and organized. Analyses and results make sense, globally. Considering the available information I do not see any serious issue with the study, but the Methods section should be expanded to clarify a few points of the data analysis.

More specifically, my concerns are the following:

Thank you for your comments. Please find our specific responses below, with line numbers used in the “simple markup” Word document format.

1) The abstract lacks results. It is written like the beginning of an Introduction, or like a longer abstract that has been trimmed. It would be better if it was more condensed and synthetic, in order to lave place to mention the key outcomes and results from the study. This would give more visibility to the article.

Thank you for your suggestion. We have revised our abstract accordingly to include summary of major findings:

Lines 26-42:

Due to the limited availability of functional data for the equine genome, as well as the technical limitations of short-read RNA-seq, existing annotation of the equine genome contains limited information about important aspects of gene regulation, such as alternate isoforms and regulatory elements, which are either not transcribed or transcribed at a very low level. To solve above problems, the Functional Annotation of the Animal Genomes (FAANG) project proposed a systemic approach to tissue collection, phenotyping, and data generation, adopting the blueprint laid out by the Encyclopedia of DNA Elements (ENCODE) project. Here we detail the first comprehensive overview of gene expression and regulation in the horse, presenting 39,625 novel transcripts, 84,613 candidate cis-regulatory elements (CRE) and their target genes, 332,115 open chromatin regions genome wide across a diverse set of tissues. We showed substantial concordance between chromatin accessibility, chromatin states in different genic features and gene expression. This comprehensive and expanded set of genomics resources will provide the equine research community ample opportunities for studies of complex traits in the horse.

2) Methods are not sufficiently detailed. For reproducibility and clarity purpose, the data analysis part of the methods section needs more information, including in particular the version of the tools and the parameters that were used.
A few examples in the "Transcriptome assembly" part:
- line 476: "Clustered transcripts were aligned to the reference genome EquCab3 (2) using minimap2 (63)". Version, parameters?
- l478: "Filtered transcripts from each sample were then merged into a single transcriptome using Cupcake (<https://github.com/Magdoll/cDNA_Cupcake/>)". Version? Which function/script?
- l480: "and further filtered to retain only those detected in more than one sample." How? Using a Cupcake script or in-house scripts?
etc.

A simple list of tools like in l.492-493 "Data processing, visualization, and statistical analyses were performed using pandas (65), matplotlib (66), seaborn (67), scipy (68), and scikit-learn" is not acceptable. Such phrasing might be used for generic tasks like data visualization or text editing, but data processing and statistical analyses need to be precisely documented. The source code could even be provided, this is common practice.
A useful thought experiment is to imagine the same for the experimental part, for instance if instead of Supp Mat 1 & 2 a single sentence were summarizing "Sample treatments, molecular processing and sequencing experiments were performed using ThermoFisher, Qiagen and Illumina equipments". This would not be considered correct. The same applies to data analysis.

Thank you for your suggestion. We have deposited the analysis scripts in github and updated manuscript accordingly (see lines 517-518, 536-537, and 560-561):

Lines 503-504, 522-523, 546-547, : Detailed program versions, commands, parameters, and code can be obtained from https://github.com/FinnoLab/FAANG_IsoSeq.

In the interest of keeping the manuscript concise, further details were not added to the main text.

3) In particular, with the exception of the ATAC-seq pipeline, it is not always clear how replicates were handled as they are barely mentioned in the analysis part.
For instance, were the Iso-Seq reads processed for each replicate separately, per tissue or altogether? The experimental description mentions 2 replicates per tissue ("One sample per sex per tissue was selected for sequencing"). Was this information considered? It seems that 4 gtf files are available per tissue on the UCSC browser. If it is the case, how were these generated?

All Iso-seq samples were merged to generate one transcriptome (as stated in lines 499-500: “Filtered transcripts from each sample were then merged into a single transcriptome”). UCSC track hub contains four RNA-seq coverage tracks per tissue (two males and two females) generated from RNA-seq bam files. With the addition of the full analyses pipelines, this should now be clarified.

4) Another point that deserves to be clarified and detailed is the way short-read RNA-seq data was used to build the Iso-Seq annotation.
Throughout most of the text, it is presented as if only Iso-seq long read data was used to generate the FAANG annotation, while Illumina short reads were only used for expression quantification. A different strategy is mentioned in the Discussion though. Also, the Methods section describes: "Finally, the total transcriptome was filtered to remove nonsense-mediated decay transcripts, transcripts without short-read coverage support, and transcripts with a splice junction not covered by short-read RNA-seq data".
This looks like a drastic filtering. If true, it means that none of the introns from the final annotation is not supported by short-read data. This raises the question of the specific contribution of the Iso-seq to the assembly in terms of isoform discovery.

Thank you for pointing this out. We have revised the language in discussion to more precisely reflect the role of RNA-seq data in our transcriptome assembly:

Lines 332-334: “…our approach centered around high-quality full-length reads from Iso-seq and used abundant RNA-seq data to ~~refine~~ **validate** splice junction, TSS, and TTS annotation.”

Regarding the splice junction filter applied to the final transcriptome, it was justified in our view because the primary challenge when it comes to identifying AS with RNA-seq data does not lie with the identification of a single splice junction but with the combination of all junctions across an entire transcript. Therefore, we reasoned that any real isoform should have sufficient RNA-seq coverage at each splice junction.

I believe that the authors should describe more precisely what they have done and what the outcome was. In particular, how many transcripts were filtered at each step of the analysis.

Thank you for you suggestion. We have added number of transcripts filtered at each step:

Lines 496-497: “5,546, 7,262, and 12,900 transcripts were removed by each filter, respectively”

The choice of ignoring the available short-read RNA-seq data to build the transcriptome using exclusively the Iso-seq data might seem a bit surprising and/or unusual. The difference between the proper pairs ratios in Sup Fig1 vs. Fig2 suggests that a fair amount of valuable information from short read data is missing from the Iso-Seq annotation. Nevertheless, this choice is not a serious issue to me as long as it is clearly presented and documented.

Thank for pointing this out. We have updated discussion to include this drawback:

Lines 359-364: “Lastly, since RNA-seq data was only used to validate and quantify transcripts identified in Iso-seq data, our approach heavily relied on Iso-seq data being complete. This unfortunately was not true as it was evident in Figure 2 and Supp Figure 1 that a substantial amount of reads from RNA-seq failed to map to Iso-seq transcriptome. A closer examination of RNA-seq data in conjunction with the Iso-seq data could further refine the FAANG transcriptome.”

Still, using short read RNA-seq data to validate the Iso-seq assembly like shown in Fig 1D is more problematic to me if this RNA-seq data has already been used to filter the annotation. Not very surprising to get more properly paired reads on Iso-seq given that isoseq transcripts with low coverage have been previously discarded, which was not done for refseq or ensemble annotations. To complement this analysis it could be informative to also provide the proportion of each annotation (ensembl, refseq and isoseq) that is not covered by RNA-seq reads.

We believe that this comparison is acceptable for two reasons:

First, while it is true that RNA-seq data from same nine tissues were used to filter Iso-seq transcripts, the same pattern was observed in most of the remaining 48 tissues, which were not used for filtering.

Secondly, filtering should decrease % properly paired reads in theory as transcripts that RNA-seq reads could have mapped to were removed and therefore the conclusion should still be valid even though reference transcriptome did not undergo the same filtering process.

5) Differential analyses of gene expression or chromatin accessibility shown in Fig 5 are not described. Required details include software tools, versions, parameters, number of tested genes/peaks, # of replicates, normalization method, model, statistical test, p-value correction, etc.

Thank you for pointing this out. We have added a method section to describe differential analyses:

Lines 549-555:

“Differential Accessibility and Expression Analyses

DAR and DEG were analyzed with similar approaches. First, a matrix of raw counts was constructed containing number of transposition events (DAR) or RNA-seq reads (DEG) for each union open chromatin peaks (DAR) or gene (DEG). The raw counts were then normalized and fitted to a negative binomial generalized linear model using DESeq2 (1.30.1) (80) package with default parameters. Wald tests were applied to obtain p-values for each region (DAR) or gene (DEG) and Benjamini-Hochberg procedure were used to control false discovery rate at 5%.”

6) Likewise, chromatin loop prediction should be described in the Methods, better than just "as described by [ref]" l. 284.
Line 289: how are loops "merged"? Given that loops seem to be considered as intervals, is a union performed, an intersection? Would this merging lead to much wider loops, does it ensure that resulting loops still have CTCF peaks at their extremities? L.295, how are loop quantified exactly? Are the read counts from both CTCF peaks added or only the distant one? Which correlation is computed (Pearson, Spearman)? Are raw read counts considered from each sample?

We added a method section to further clarify the details:

Lines 588-606:

“Chromatin Loop Prediction and CRE-Gene Interaction Analyses

To obtain a pan-tissue set of chromatin loops in absence of chromatin interaction data, CTCF ChIP-seq data was used to predict chromatin loops in each tissue, using the algorithm proposed by Oti et al.(48). Briefly, CTCF ChIP-seq peaks were identified using MACS3 (42) (-q 0.05 -f BAM -g 2365156725 --keep-dup auto). CTCF motifs were then extracted from these peaks using FIMO function from MEME (85) (--motif MA1930.1 --parse-genomic-coord). Then in each tissue, each chromosome was scanned continuously, and a loop was recorded by detecting a pair of parallel and anti-parallel CTCF motifs. The predicted CTCF loops across tissues were then combined by merging overlapping loops using bedtools (78) merge function.

To obtain a list of CREs, neighboring active chromatin states (states 2-5 and 8-11) were merged and annotated by their proximity to a known gene (genic, intergenic, and TSS-promoter) as well as their closest genes. These CREs were then quantified by number of overlapping reads from their corresponding H3K27ac ChIP-seq data. Reads counts were normalized by a scaling factor calculated using weighted trimmed mean (TMM) method. Normalized RNA-seq read counts were obtained from previous analysis (see Methods - Differential Accessibility and Expression Analyses). Spearman correlation between H3K27ac read count in each CRE and RNA-seq read count in each gene was calculated using spearmanr function from SciPy (68).”

Before considering the correlation between the CRE and the gene expression, wouldn't it make sense to check the chromatin correlation between the promoter vs. CRE peaks of each loop?

In our analysis, CREs include promoter regions as some promoters have been shown to behave in an enhancer-like manner by interacting with other promoters. Therefore, we did not consider it to be particularly informative to correlate promoters with themselves.

Supp Fig 5 is interesting. It is surprising to see such a strong downstream trend. Difficult to estimate if there is a normalization bias. What is the resulting figure when considering all the predicted promoter loops before the correlation filter?

Without filtering, CRE-gene pairs simply include all combinations of CREs and genes found within each chromatin loop. This in our opinion is not an informative figure, as it would simply show the average distribution of CRE-gene distance within each chromatin loop.

7) Regarding transcript-to-gene ratio in horse vs. human, the authors claim l. 79 that "This difference is largely due to the limitation of the short-read sequencing technology". This is not true. Human or mouse genome annotations have always had more isoforms per gene compared to non-model species, even before long read RNA-seq, and even before RNA-seq technologies altogether. The main reason for a deeper annotation of the human transcriptome is the difference in transcriptomic data quantity. What is currently called "short-read" RNA-seq is not to blame for discovering less isoforms. By the way, the two references that are cited at the end of the sentence do not support that statement. Similarly, the statement "short-read sequencing-based RNA-seq data meant that alternate alternate isoforms could not be accurately resolved" (abstract, l35) is too extreme and should be softened because the current phrasing implies that accurate identification of an isoform is impossible with RNA-seq, which is misleading. Full length isoforms have been correctly identified using "short-read" RNA-seq and confirmed by Sanger sequencing for instance.

Thank you for pointing this out to us. We have revised accordingly to more accurately describe the current challenges:

Lines 76-79: “While this difference can be attributed to vast quantities of transcriptomic data available in human, recent developments in the long-read sequencing technology provided a unique opportunity for non-model organisms to quickly annotate AS without generating a prohibitively large amount of data.”

Additionally, the abstract has been revised accordingly.

8) l. 292: "This was comparable to...". Chromatin loops and TADs are distinct 3D structures, with different scales. This comparison is not really relevant.

We have removed this sentence.

9) Is the global FAANG annotation file available somewhere? Maybe this gtf file could be provided as Supplementary Material for an easy access.

We updated data access statement to include a repository of data underlying UCSC genome browser track hub. A gff3 file is also included as a supplementary file.

10) At the beginning of the CRE section l.228 it would be better to cite the Kingsley et al reference again to remind the readership that the histone data was generated in a previous study, otherwise it is a bit confusing.

Thank you for your suggestion. We have revised accordingly:

Lines 281-283: “we predicted chromatin loops and correlated H3K27ac signal (data originally generated and reported by Kingsley et al.(42)) of CREs with expression of genes resided within the same chromatin loops.”

Discussion
- The authors mention monoexonic transcripts: what is the proportion of those in the Iso-seq assembly?

We added to the result the number of multi-exonic transcripts in Iso-seq transcriptome:

Lines 130-131: “A majority of these transcripts (51,639) were multi-exonic.”

- l 332: "our approach used abundant RNA-seq data to refine splice junction, TSS, and TTS annotation." This is not what I saw in the Methods.

We have revised the language in discussion to more precisely reflect the role of RNA-seq data in our transcriptome assembly:

Lines 332-334: “…our approach centered around high-quality full-length reads from Iso-seq and used abundant RNA-seq data to ~~refine~~ **validate** splice junction, TSS, and TTS annotation.”

- l 343: "While an aggressive approach was taken to ensure 5’ completeness". Which one?

We revised the language for clarity:

Lines 343-345: “While an aggressive approach was taken to ensure 5’ completeness by collapsing transcripts that only differ at 5’ ends”

- Just to comment about the DAR/DEG discrepancy in Q2 & Q4, it is true that the dynamic range difference between RNA and DNA quantities is an important factor to consider. Other possible explanations include the time difference between the regulatory action on the DNA and the subsequent effect on the transcriptome, or the simple fact that CREs do not only include enhancers but inhibitors too.

Thank you for the suggestion. We have updated the discussion to include these possible explanations.

Lines 394-395: Additionally, the presence of silencers in CREs or the time difference from CRE activation to change in mRNA abundance may also explain the observed discordance.

- If the article needs to be shortened, the last paragraph of the Discussion could easily be removed as there is no surprise to find "substantial cell-type differences" between tissues, in particular brain and heart, or testes vs. the others.

We intended to point out the sub-population structure within each tissue sample as suggested by DAR vs DEG analyses rather than population differences between tissue types. We have revised the text for clarity:

Lines 438-439: “…suggest substantial sub-population differences within each of these tissues. ”

Figures
- Shouldn't the units be between parentheses? For instance "Distance to Ensembl TTS (bp)" instead of "Distance to Ensembl TTS, bp".

- Fig 1A: Since the Iso-seq read mapping/processing does not seem to use a reference annotation, how are Iso-seq transcripts assigned to Ensembl TTS? How is it possible to know that the ones several Kb downstream are from the same gene?

While the assembly did not rely on a reference transcriptome, it was later compared against RefSeq transcriptome to classify each transcript by their proximity to a reference transcript. We have updated the methods for clarity:

Lines 492-493: “SQANTI3 (61) was then used to classify and annotate the transcriptome against the RefSeq transcriptome as reference.”

- Fig 1B: x-axis label would be more clear with "downstream vs. upstream" or "downstream/upstream ratio" instead of "up- and downstream".

Thank you for the suggestion. The figure was edited accordingly.

- Was the Fig 1D analysis performed by mapping all the short-read RNA-seq of the entire project each time, including the 57 tissues x 4 replicates, or just a subset?

It was performed for all 57 tissues across 4 replicates (2 for reproductive tissues). We updated figure legend for clarity:

Lines 643-646: “(D) Distribution of mapping rates against Iso-seq transcriptome vs. Ensembl or RefSeq transcriptome across 57 RNA-seq samples”

- Fig. 3B: the figure does not support the statement from the text ("Area under curve (AUC) values of at least 0.6 were achieved for all tissues evaluated (Figure 3B).") because only liver is displayed. Maybe it is possible to show the nine selected tissues, a distribution of the AUC values, or to change the sentence.

Figure 3B was used as a representative figure. We have added an additional supplemental figure (Supp Figure 2) to show AUC values of all eight tissues that were examined here.

Typos:
l.179: "Accuracies of peak calling were using published" [estimated]?

Thank you for pointing this out. We have fixed the typo:

Line 179-180: “Accuracies of peak calling were estimated using published histone ChIP-seq data from the same tissues”

l. 332: "long isoform sequencing" long read?

We used long isoform sequencing to specifically refer to application of long-read sequencing in RNA

Reviewer #3: This is a well-written manuscript that reports a comprehensive annotation of the gene regulatory landscape in the horse genome. The annotation resources will be especially valuable to the equine research community, and will also be of broader interest in the area of mammalian gene regulation. The paper is original and does not duplicate previous work. Literature is appropriately cited. The methods are sound and sufficiently described, with some detailed protocols provided in the supplemental material. Original data is deposited in appropriate repositories, and the study conforms to relevant guidelines. The study is very thorough, and I do not have any suggestions for additional analyses.

Thank you for your comments. Please find our specific responses below, with line numbers used in the “simple markup” Word document format.

Minor Comments:
The data and analyses fully support the claims, however not all the numerical data that underlies figures are provided in spreadsheets as supporting information. The only spreadsheets provided are for the GO enrichment and motif enrichment.

Unfortunately, most of this numerical data that is presented in our graphs is difficult to include in table format and does not necessarily add to the presentation of results (i.e. coverage ratio, histogram of distance to TSS, mapping rates of 200+ samples, etc). However, the authors are willing to provide any of this graphed numerical data upon request.

Figure 1A and 1B: It would be helpful to state in the Figure legend what FSM and ISM are (even though it’s in the text).

Thank you for your suggestion. We have revised accordingly:

Line 643-646: “(D) Distribution of mapping rates against Iso-seq transcriptome vs. Ensembl or RefSeq transcriptome across 57 RNA-seq samples. FSM: full-splice match; ISM: incomplete-splice match; TTS: transcription start site; TSS: transcription termination site”

Figure 4: There is a misspelling “Uniquitous”

Thank you for pointing out this typo. We have revised accordingly. See Figure 4

Supplementary Figure 2 Legend: It seems backwards. Since most of the changes are positive, should it be “Changes in percentages of properly paired reads aligned to Ensembl or RefSeq transcriptomes compared to Iso-seq transcriptome”.

This has been revised to Supplementary Figure 1. Since the use of the combined Iso-seq transcriptome increased the percentage of properly paired reads, we used the verbiage to compare Iso-seq vs. Ensembl/RefSeq.

Figures S3 and S4: The explanation of the part with the dots is not clear.

We have revised the figure legend for clarity:

Lines 705-706 and 713-714: “Bottom right: each column denotes a unique intersection set of peaks wherewith filled dots indicating that segments peaks in this intersection set were found in the corresponding tissue”

The resources generated would be even more valuable to the research community if the new predicted gene set, or at least the Iso-seq predicted gene set, would be available in gff3 or gtf format, and fasta formatted sequences for transcripts and coding sequence would be made available. I realize the genes are available in BigBed format via UCSC, but converting it to gff3 or gtf might not be straightforward for everyone. Could these files be provided in a data archive site such as Figshare?

We updated data access statement to include a repository of data underlying UCSC genome browser track hub. A gff3 file is also included as a supplementary file.

**Have all data underlying the figures and results presented in the manuscript been provided?**
Large-scale datasets should be made available via a public repository as described in the *PLOS Genetics* [data availability policy](http://journals.plos.org/plosgenetics/s/data-availability), and numerical data that underlies graphs or summary statistics should be provided in spreadsheet form as supporting information.

Reviewer #1: Yes

Reviewer #2: Yes

Reviewer #3: **No:** Not all the numerical data that underlies figures are provided in spreadsheets as supporting information, but it might not be feasible to provide all of it in spreadsheets.
